# Distinct timescales for the neuronal encoding of vocal signals in a high-order auditory area

**DOI:** 10.1038/s41598-021-99135-w

**Published:** 2021-10-04

**Authors:** Aurore Cazala, Catherine Del Negro, Nicolas Giret

**Affiliations:** Université Paris-Saclay, CNRS, Institut des Neurosciences Paris-Saclay, 91400 Saclay, France

**Keywords:** Cortex, Sensory processing, Cellular neuroscience, Perception, Action potential generation

## Abstract

The ability of the auditory system to selectively recognize natural sound categories while maintaining a certain degree of tolerance towards variations within these categories, which may have functional roles, is thought to be crucial for vocal communication. To date, it is still largely unknown how the balance between tolerance and sensitivity to variations in acoustic signals is coded at a neuronal level. Here, we investigate whether neurons in a high-order auditory area in zebra finches, a songbird species, are sensitive to natural variations in vocal signals by recording their responses to repeated exposures to identical and variant sound sequences. We used the songs of male birds which tend to be highly repetitive with only subtle variations between renditions. When playing these songs to both anesthetized and awake birds, we found that variations between songs did not affect the neuron firing rate but the temporal reliability of responses. This suggests that auditory processing operates on a range of distinct timescales, namely a short one to detect variations in vocal signals, and longer ones that allow the birds to tolerate variations in vocal signal structure and to encode the global context.

## Introduction

Vocal communication signals can provide a richness of information through both their acoustic structure and subtle variations in their acoustic features^1,2^. The same word spoken by different people conveys information about the intended meaning through an invariant acoustic structure among uttered signals. Fine variations in the temporal and acoustic features between different individuals may also provide information about the gender, the emotional state, and the identity of the emitter. Vocal communication is therefore a computational challenge, requiring the auditory system to selectively extract invariant information with a certain degree of tolerance towards variations for categorization while maintaining a sufficient sensitivity to variations to be able to extract supplementary information^3^. Within this framework, it is still largely unknown how this balance between tolerance and sensitivity to variations in acoustic signals is coded at a neuronal level^4–6^.

Songbirds offer a powerful model to explore the neural coding principles that underlie this balance. Birdsong is a complex multi-cue signal that is particular to each species and exhibits subtle variations that may carry information such as group or individual identity, emotional or motivational state, or physical condition^7,8^. Among the different songbird species, the zebra finch is well suited to investigate how subtle variations in their highly repetitive communication sounds are encoded within the auditory system. The male zebra finch typically produces a highly specific but at the same time stereotyped song motif that differs between individuals and includes several distinctive sound elements, called syllables, that are always produced in the same order^9^. Despite the high level of stereotypy in their acoustic structure, motifs vary between renditions and these variations can convey information about the social context, *e.g.,* the presence or absence of females^10^, and a recent study has provided evidence that zebra finches are capable of perceiving these subtle variations^11^. Male zebra finches repeat their song many times per day and this repetition of the same stimulus is well known to elicit habituation in behavioral and neural responses, which in turn raises the question of whether variations in the stimulus could elicit changes in the listeners neural responses.

In songbirds, the processing of complex, behaviorally relevant acoustic signals, including calls and songs, involves the caudomedial nidopallium (NCM), an auditory area that is analogous to the secondary auditory cortex in mammals. The NCM thus represents a good candidate to investigate how the balance between tolerance and sensitivity to subtle variations in acoustic signals is encoded^3^. Neurons in this auditory area display a clear preference for natural over artificial sounds. Regarding conspecific vocal signals, they may exhibit invariant responses to call categories^12,13^. Despite this tolerance to variations in vocal signals, neurons in the NCM are capable to facilitate recognition of familiar vocalizations that only exhibit subtle acoustic differences between categories^14–16^. Neurons in the NCM also display stimulus-specific adaptation such that repeated exposure to a given auditory stimulus induces a decrease in the response while exposure to a novel stimulus or to the same stimulus with a different order of the sound elements resets the response^15,17–20^. To date, this phenomenon, interpreted as reflecting memory formation, has only been reported in experiments in which the exact same sound stimuli were repeatedly presented. However, in the wild, individuals are never exposed to identical vocal signals as fine natural variations in acoustic features always occur between renditions, raising the question of whether these variations might affect the intensity and time course of neuronal responses in the NCM. Based on extracellular recordings in both anesthetized and awake zebra finches, we found that sequences of song elements that either varied in acoustic detail or remained the same between renditions differentially impacted the temporal reliability of the neuronal responses. This impact in spike timing was observed using a high temporal resolution that allows for the temporal integration of acoustic features over different time scales.

## Results

**Figure 1:**
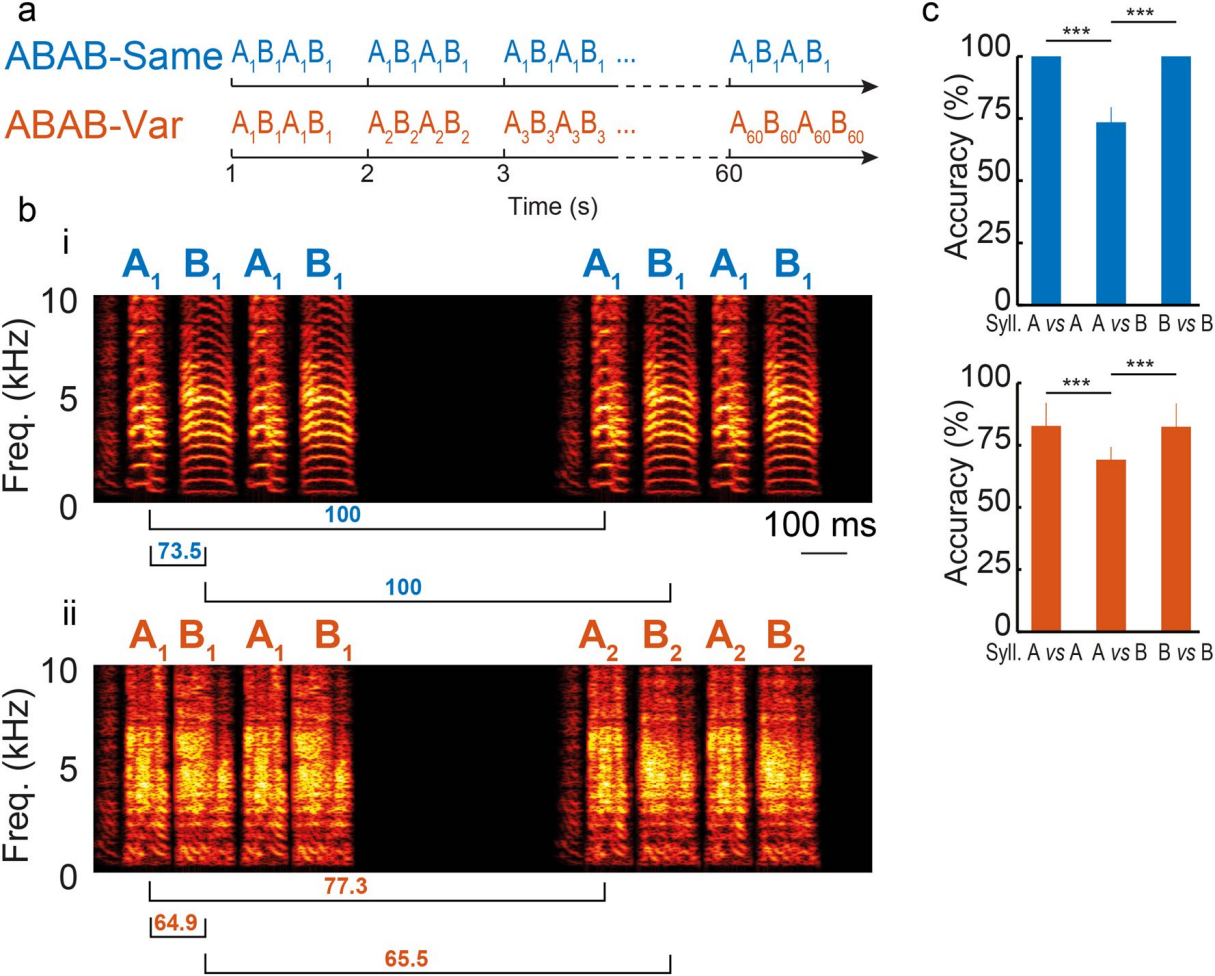
A single sequence or sequences with natural variations found in individual’s songs were used to build two series types: ABAB-Same and ABAB-Var series. (**a**) Schematic diagram of the structure of ABAB-Same (top) and ABAB-Var (bottom) series. A and B depict two syllable types used to form ABAB sequences. The ABAB-Same series consisted of 60 repetitions of a single ABAB sequence while the ABAB-Var series consisted of 60 distinct renditions of a given ABAB sequence. These renditions called sequence “variants” were labelled as A_n_B_n_A_n_B_n_ (n varying from 1 to 60). A_n_ and B_n_ were distinct exemplars of a single syllable type that were extracted from the song’s repertoire of a given individual. Each sequence was presented at a rate of one per second. (**b**) Example spectrograms of two consecutive sequences within an ABAB-Same (i, no variants) and ABAB-Var (ii, variants) series. Note the subtle changes between A_1_B_1_A_1_B_1_ and A_2_B_2_A_2_B_2_ sequences of the ABAB-Var serie (e.g. power at ~ 5 kHz on syllable B. Underneath each spectrogram are the accuracy scores (%) computed with SAP 2011^21^ (see main text for further details) between A and B syllables across the two successive example renditions of the ABAB-Same and ABAB-Var sequences. (**c**) Mean (+ /- STD) of the accuracy scores computed between A and B syllables across the 60 renditions of all the ABAB-Same (top) and ABAB-Var (bottom) sequences. *** *p* < 0.001.

To explore the neuronal sensitivity to subtle acoustic variations between renditions of vocal signals in a high-order auditory area, we performed extracellular recordings of NCM neurons in adult male zebra finches that were either awake or anesthetized (n = 4 and n = 7, respectively) while playing back sequences assembled using song syllables from one individual. These sequences were arranged in two different sound series, henceforth referred to as the ABAB-Same and ABAB-Var series. They consisted of two song syllables, named A and B, that were repeated twice in alternating order to form the ABAB sequence. The ABAB-Same series was built from 60 repetitions of a single ABAB sequence while the ABAB-Var series contained 60 natural variants of a given ABAB sequence (Fig. 1a–c). Both series were presented to each recording site in a random order (*i.e.*, ABAB-Same was played either before or after ABAB-Var). The acoustic similarity between sequence variants of the A and B syllables was evaluated using the percent accuracy score in Sound Analysis Pro 2011^21^. The average similarity of renditions of the A and B syllables in the ABAB-Var sequence was 83.2% and 81.9%, respectively. In contrast, the similarity between the A and B syllables within a given sequence was significantly lower, namely 73.5% in the ABAB-Same sequence and 68.8% (on average) in the ABAB-Var sequence (t-tests, *p* < 0.001; Fig. 1c).

### Acoustic variations elicit no changes in response strength, neither in awake nor anesthetized birds

Although syllables in zebra finch songs contain acoustic variability between renditions, it remains unclear whether NCM neurons are sensitive to these natural variations. Auditory responses of NCM neurons in zebra finches are known to decrease as they get exposed to repetitions of the same songs or calls^15,17,19,20,22^. Here, we hypothesized that if NCM neurons are sensitive to these variations, this repetition-based decrease in neuronal responses could be impacted by acoustic variability. Nevertheless, NCM neurons may possess some tolerance towards a certain level of acoustic variability which would then be reflected by a similar decrease in the response with each repetition, regardless of whether they vary acoustically.

**Figure 2:**
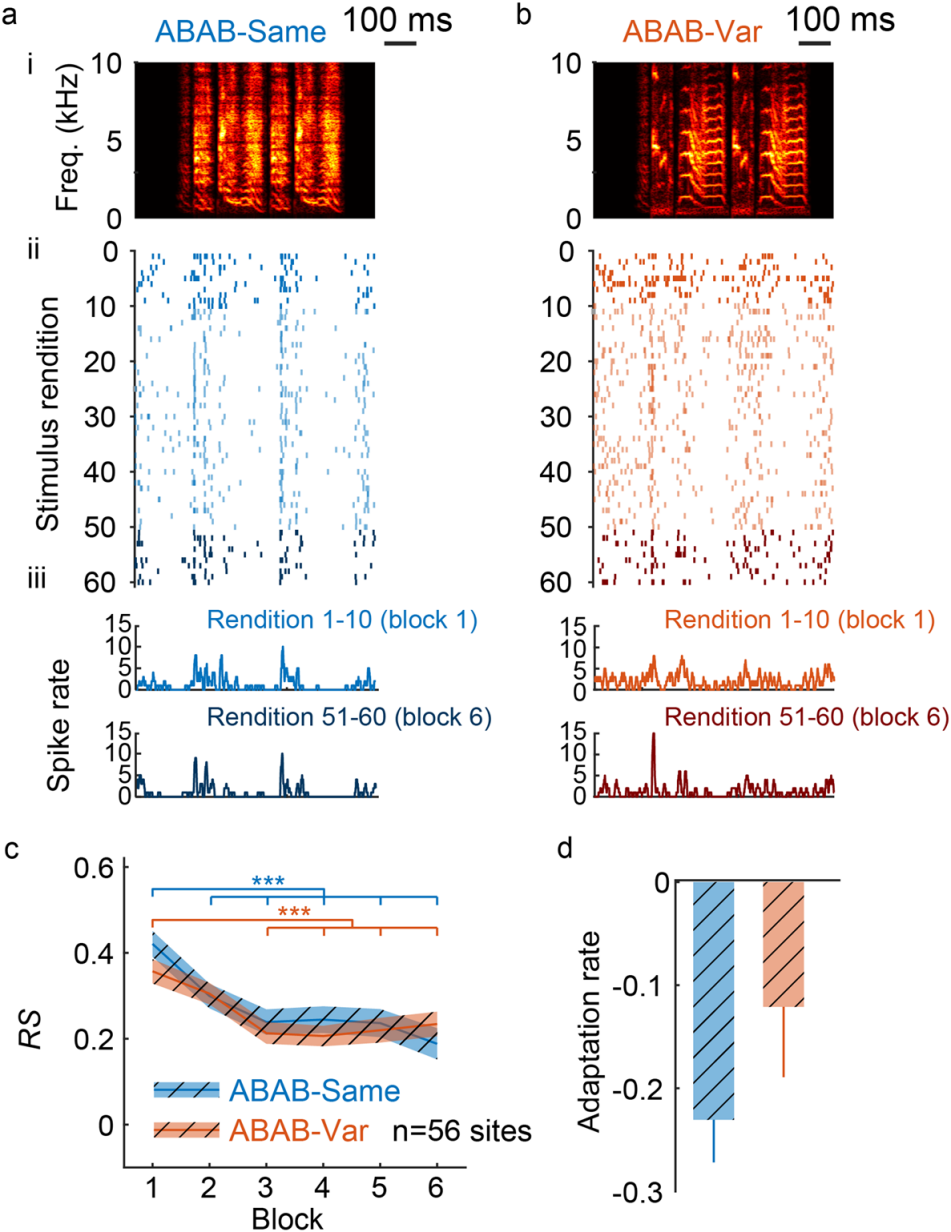
Auditory responses to 60 repetitions of a single sequence (ABAB-Same series) and to 60 sequence variants (ABAB-Var series) in awake birds. Responses of a representative unit to the ABAB-Same (**a**) and the ABAB-Var (**b**) series used as auditory stimuli. Neuronal responses that are time-aligned with sequence spectrograms (ai and bi: the sequence repeated 60 times for the ABAB-Same example series and one sequence variant for the ABAB-Var example series) are shown as raster plots (aii and bii, 60 iterations, dark colors for the 10 first and 10 last trials) and peristimulus time histograms (aiii and biii; 10 ms bin width; for the 10 first (top) and the 10 last (bottom) trials). (**c**) Modulation of responses over the six successive blocks of ten trials (each block for the ABAB-Var series includes 10 variants of the auditory sequence). The *RS* values estimated the strength of the responses driven by the series used as auditory stimulus. Thick line indicates mean responses for the population of recording sites (n = 56). Hatched area represents SEM. (**d**) Adaptation rate (mean ± SEM) of responses computed over the 10 first trials did not significantly differ between the two series.

To address this issue, we performed chronic recordings in four awake birds with three (range 2–5) recording sessions (3.6 electrodes per recording session, range 2–7) per bird with a 4.5 day gap (range 1–9) between two successive recording sessions. These recording sessions took place in a sound-attenuation chamber and birds were restrained for the duration of each session (see “Methods” for further details) to minimize movement artefacts. We analyzed the spiking activity at 56 recording sites, whose locations ranged from the dorsorostral (maximal depth 2000 µm) to the dorsocaudal portions. They were driven by the playback of the ABAB-Same and ABAB-Var sequences (Fig. 2a,b).

To examine whether the time course of auditory responses differed between the ABAB-Same and the ABAB-Var series, we performed a repeated measures (RM) ANOVA on the response strength (*RS*), computed from firing rates averaged over the entire sequence duration, using a linear mixed-effect model with sequence type and block repetition as cofactors and units as a random factor (Fig. 2c). We use the term “block” because data were averaged over 10 trials. All trials were performed using the same frequency of 1 s^−1^ for both series. Results indicated that response strength did not differ between ABAB-Same and ABAB-Var series (sequence type factor; F_1, 564_ = 0.03, *p* = 0.85). The RM ANOVA revealed an effect of block repetition factor on *RS* values (F_5, 564_ = 30.38, *p* < 0.0001) with a decrease in response strengths for both series (post-hoc tests: ABAB-Same: block 1 *vs*. block 2 to 6 all *p* < 0.001; ABAB-Var: block 1 *vs*. block 3 to 6, all *p* < 0.001). Statistical analysis also revealed a significant interaction between block repetition and series type factors (F_5, 564_ = 2.26, *p* = 0.047) suggesting that the time course of auditory responses over the 60 renditions of ABAB sequences depended on whether the acoustic features of syllables varied. Responses change dramatically during the initial stimulus presentations^22^. Here, NCM neurons displayed a significant decrease in their activity between the first and second block of the ABAB-Same series. We therefore tested whether NCM neuronal responses adapted more rapidly to the ABAB-Same series compared to ABAB-Var. The adaptation rate, calculated as the slope of the linear regression over the 10 first stimulus renditions for each unit^17,23–25^, was indeed higher for ABAB-Same (Fig. 2d), although the difference was not statistically significant (t_1, 55_ = 1.18, *p* = 0.24). Hence, there was no clear effect of inter-rendition acoustic variations in syllable features on the time course of neuronal responses.

**Figure 3:**
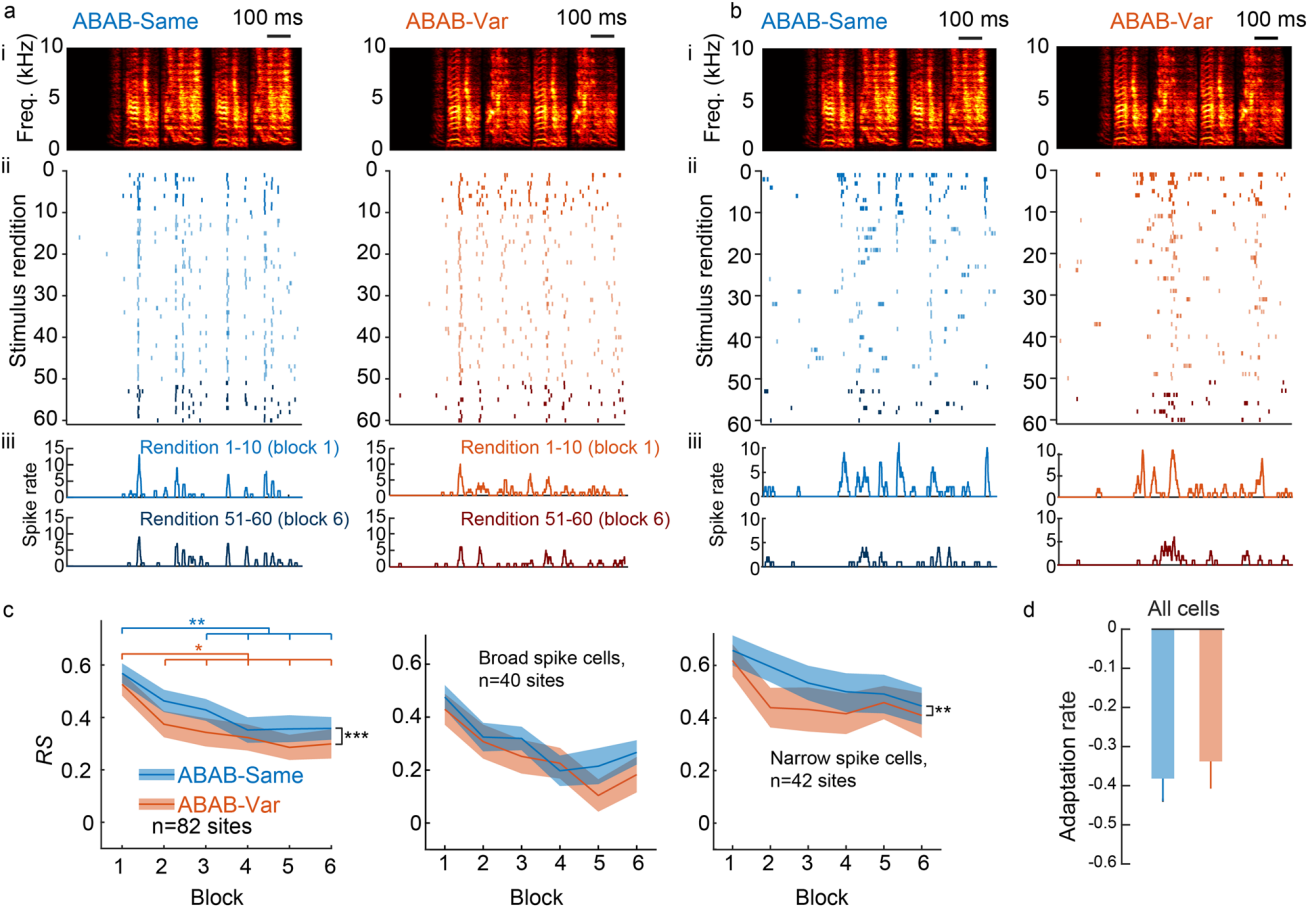
Auditory responses in anesthetized birds. From rendition to rendition, spike timing greatly changed when sequence variants (ABAB-Var series) were played back. No such changes were observed when the same sequence was repeated (ABAB-Same series). (**a**,**b**) Neuronal responses of representative putative narrow spike (**a**) and broad-spike (**b**) cells to playback of ABAB-Same (left panels) and ABAB-Var (right panels) series (spectrogram on top, ai: the sequence repeated 60 times for the ABAB-Same example series and one sequence variant for the ABAB-Var example series) are shown as raster plots (aii and bii, 60 iterations, dark colors for the 10 first and 10 last trials) and peristimulus time histograms (aiii and biii; 10 ms bin width; for the 10 first (top) and the 10 last (bottom) trials) that are time-aligned with sequence spectrograms. (**c**) ABAB-Same series evoked higher responses (*RS* values) than ABAB-Same series at the population level (left) and for the sub-population of narrow spike cells (right), but not for broad spike cells (middle). Thick line indicates mean values and shaded area represents SEM. (**d**) Despite of the difference in the response strength, adaptation rate computed over the first ten stimuli presentations, thus indicated that response strength similarly changed with repeated exposure to sequences. Thick line indicates mean values; shaded area represents SEM. Significant difference: *p < 0.05, **p < 0.01, ***p < 0.001.

We collected responses to single units in order to assess them with regard to the different cell types. For this purpose, we collected extracellular recordings in NCM in seven isoflurane-anesthetized adult males, selecting only well-isolated responsive single units (n = 82, example unit on Fig. 3a,b). These single units were placed at locations ranging from the dorsorostral (maximal depth 1900 µm) to the dorsocaudal portions (Cazala et al.^20^).

The analysis revealed that response strength, averaged over the six blocks, differed between ABAB-Same and ABAB-Var (RM ANOVA performed on *RS* values; series type factor: F_1, 1055_ = 12.87, *p* = 0.0003). However, auditory responses did not differ in block-by-block comparisons (post-hoc tests; all *p* > 0.64). As in awake birds, neuronal responses showed the well-described adaptation to block repetitions for both series (Fig. 3c; block repetition factor: F_5, 1055_ = 13.02, *p* < 0.0001; post-hoc tests: ABAB-Same series: block 1 *vs*. block 3, 4, 5 and 6, all *p* < 0.01; ABAB-Var series: block 1 *vs*. block 2, p < 0.0021, block 1 *vs*. block 3, 4, 5 and 6, *p* < 0.01) with no difference in adaptation rate during the first ten trials (F_1, 81_ = 0.74, *p* = 0.46). Therefore, subtle variations in the acoustic features of syllables in the ABAB-Var series had no discernible impact on the neuronal firing rates.

Two cell types can be distinguished in NCM^3,20,26–28^. Responsive NCM neurons were split into two populations according to the peak-to-peak width of their action potential: neurons with broad spikes (≥ 0.3 ms; *n* = 40, width = 0.49 ± 0.10 ms) and neurons with narrow spikes (< 0.3 ms; *n* = 42, width = 0.27 ± 0.07 ms). The RM ANOVA performed on *RS* values according to the block repetition revealed a significant decrease in response strength of both cell types (Fig. 3c; broad-spike cells, linear-mixed effect: F_5,428_ = 9.29, *p* < 0.0001; narrow-spikes cells, linear-mixed effect: F_5,448_ = 5.01, *p* < 0.0003) and a significant series type effect for narrow-spiked cells (broad-spike cells, series type factor: F_1,428_ = 3.10, *p* = 0.08; narrow-spikes cells, series type factor: F_1, 448_ = 7.72, *p* < 0.006), but no significant interaction between the two factors in either cell type (broad-spike cells, F_5,428_ = 0.53, *p* = 0.75; narrow-spikes cells, F_5,448_ = 0.55, *p* = 0.73). When the analysis focused on the first ten renditions of the first block only, neither cell type exhibited any changes in adaptation rate in response to natural variations (Fig. 3d; broad-spike cells, paired t-test: t_38_ = 1.39, *p* = 0.17; narrow-spike cells, paired t-test: t_40_ = 0.26, *p* = 0.79; note that for both cell types, one unit was removed because it did not spike during the first trial).

### Impact of acoustic variations on spike-timing reliability

**Figure 4:**
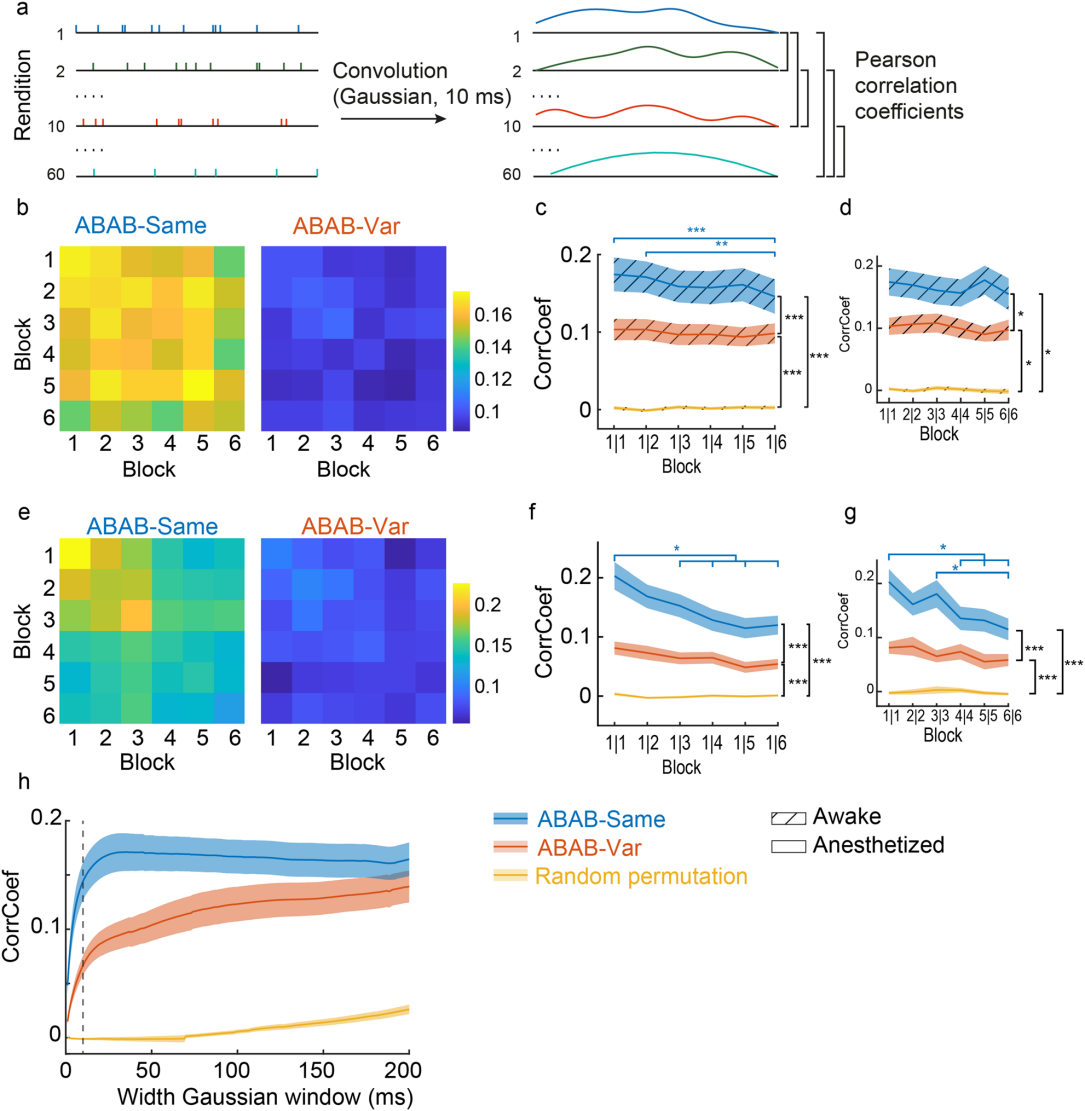
Reliability of spike trains in awake and anesthetized birds. (**a**) To compute the reliability of the spike trains (CorrCoef), a convolution of spike times with a Gaussian window of 10 ms was performed. Pearson correlation coefficients were computed on convoluted spike trains between two renditions. CorrCoef values within a block, e.g. block 1 *vs* 1 (1|1), correspond to the mean of the Pearson correlation coefficients between renditions one to ten. CorrCoef values between blocks, e.g. block 1 *vs* 2 (1|2), correspond to the mean of all the Pearson correlation coefficients between renditions one to ten and eleven to twenty (*i.e.* rendition 1 *vs* 11 to 20, rendition 2 *vs* 11 to 20, etc.). Reliability of spike trains in awake (**b**) and anesthetized (**e**) birds illustrated by heatmaps (right: ABAB-Same series; left: ABAB-Var series). Spike-trains reliability, quantified by the CorrCoef index, was lower when sequence variants were presented. Blue color indicates low CorrCoef values. At the population level in awake (**c**,**d**) and anesthetized (**f**,**g**) birds, differences in spike-timing reliability and in its time course between the two series. CorrCoef values were computed from spike trains evoked by the first ten trials and those evoked by the ten trials of the six blocks (block 1 to 6, **c**, **f**) and within each block (**d**,**g**). In both awake and anesthetized birds, CorrCoef (mean ± SEM) changed with stimulus exposure when the same sequence (blue line) was repeated while it remained similar when sequence variants (red line) were played back. CorrCoef values were higher than those of spike trains in which spike timing was randomly permutated (yellow line). (**h**) Varying the Gaussian window width used to compute the convolution of spike trains from 1 to 200 ms affects CorrCoef values. In the present study, a 10 ms Gaussian window width to compute CorrCoef values (vertical dashed line) and CorrCoef values differed between the two series. No difference between ABAB-Same and ABAB-Var was observed when the time window exceeds 98 ms. CorrCoef values were also computed on spike trains after a random permutation of the spike timing. Significant difference: *p < 0.05, **p < 0.01, ***p < 0.001 (see main text for statistics details).

The response strength index was estimated over the entire duration of sound sequences. To assess the potential impact of the acoustic variability of syllable features on neuronal responses at shorter timescales, we analyzed the temporal pattern of responses by computing the trial-to-trial spike timing reliability coefficient, the CorrCoef (Fig. 4a). Most analyses based on CorrCoef values resulted from computations based on a 10 ms Gaussian window width (see “Methods”). High values indicate a high spike train reliability across trials while low values mean great variations in temporal patterns of spike trains. This coefficient was calculated based on responses during 20 presentations, *i.e.*, ten presentations of sequence stimuli of a given block and ten presentations of each of the 6 blocks.

Results indicated that CorrCoef varied between [− 0.07 and 0.69; average of 0.13] and [− 0.07 and 0.78; average of 0.11] in awake and anesthetized birds, respectively (Fig. 4b,e), which is in the usual range for cortical^29–31^ and NCM neurons^20^. Most results were consistent between awake and anesthetized birds and indicated differences in spike-train reliability between different series types, *i.e.*, depending on whether acoustic features of syllables varied across renditions (linear mixed effect model, RM ANOVA; awake birds: series type factor; F_2, 221_ = 63.18, *p* < 0.0001). The impact of sequence repetition also varied with series type in both awake and anesthetized birds (linear mixed effect model, RM ANOVA; awake birds: block repetition factor, F_5, 1105_ = 2.06, *p* = 0.068; series type and block repetition interaction, F_10, 1105_ = 2.80, *p* = 0.002; anesthetized birds: series type, F_2, 1869_ = 501.09, *p* < 0.0001; block repetition factor, F_5, 1869_ = 6.17, *p* < 0.0001; series type and block repetition interaction, F_10, 1869_ = 4.27, *p* < 0.0001). The trial-to-trial spike-timing reliability was significantly lower with the ABAB-Var series (Fig. 4c,f) suggesting greater variations in the spike-timing of responses when sequences consisted of ABAB variants rather than identical ABABs. Importantly, although spike-train reliability was low for variants, this did not result from a lack of temporal organization within spike trains. In fact, the CorrCoefs of spike trains were significantly higher with variants compared to when inter-spike times were randomly distributed (awake birds; post-hoc test: ABAB-Same *vs* Random permutation, *p* < 0.0001; ABAB-Var *vs* Random permutation, *p* < 0.0001; anesthetized birds: post-hoc test: ABAB-Same *vs* Random permutation, *p* < 0.0001; ABAB-Var *vs* Random permutation, *p* < 0.0001; yellow line in Fig. 4d,g). This suggests that although the trial-to-trial reliability in spike trains varied considerably throughout the exposure to the variants a certain degree of temporal organization persisted nonetheless.

Repetition of the same sequence modulated the trial-to-trial reliability of spike trains. CorrCoefs were decreasing significantly with renditions of the ABAB-Same sequence, as indicated by post-hoc tests focused on comparisons between the first and subsequent blocks (awake birds, Fig. 4c: block 1|1 *vs*. block 1|6; *p* < 0.0001; block 1|2 *vs*. block 1|6; *p* = 0.0017; anesthetized birds, Fig. 4f: block 1|1 *vs.* block 1|3 to 1|6, multiple *p* < 0.02). In contrast, the trial-to-trial reliability of spike trains evoked by variants in the ABAB-Var series remained constantly low between blocks (awake birds, *p* > 0.97; anesthetized birds, *p* > 0.56). The gradual decrease in spike-timing reliability that occurred with the ABAB-Same series could be due to an increased variance in trial-by-trial spike-timings across sequence renditions and/or to an overall time-shift in the spike trains evoked by later blocks. To identify the underlying cause, we computed the trial-by-trial spike-timing reliability (CorrCoef) within each block and compared them between blocks (*e.g.*, block 1|1 *vs*. block 2|2 compared to 6|6, block 2|2 *vs.* block 3|3 compared to 6|6). Identical CorrCoef values between blocks would indicate a similar variance in trial-by-trial spike-timing and, consequently, an overall change in the temporal pattern of responses driven by ABAB-Same sequences in later blocks. In awake birds, CorrCoef remained stable across block renditions (Fig. 4d, linear mixed effect model, RM ANOVA; block repetition factor: F_5, 825_ = 0.52, *p* = 0.76; series type and block repetition interaction: F_10, 825_ = 0.96, *p* = 0.48) suggesting small changes in the variance of the trial-by-trial spike-timing. In anesthetized birds, CorrCoef significantly decreased with the repetition of ABAB-Same sequences (Fig. 4g, block repetition factor: F_5, 1582_ = 3.30, *p* < 0.006; series type and block repetition interaction: F_10, 1582_ = 2.53, *p* = 0.0051; post-hoc test: block 1|1 *vs* block 4|4, 5|5 and 6|6, multiple *p* < 0.02; block 3|3 *vs* block 6|6, *p* < 0.002), which suggests that in anesthetized birds both factors could influence spike-timing reliability, *i.e.*, an increase in the variance of the trial-by-trial spike-timing as well as a shift in the temporal patterns of discharges.

In order to test whether the spike-timing accuracy (CorrCoef) depended on the width of the Gaussian window (we had used 10 ms—considered optimal for discriminating between conspecific songs in auditory structures^31–33^—to perform the previous computations), we tested Gaussian window widths ranging from 1 to 200 ms. By increasing the width of the Gaussian window, spike trains are increasingly smoothed, which in turn means that the trial-to-trial reliability of spike trains increasingly depends on firing rate rather than spike timing accuracy. Our aim was to determine the temporal resolution at which spike-timing accuracy no longer differed between the ABAB-Var and ABAB-Same series. While CorrCoef values of ABAB-Same series plateaued for a Gaussian window width of about 10 ms, those of ABAB-Var series continuously increased but remained lower for widths up to 170 ms (Fig. 4h). Both values are always considerably higher compared to values for a random permutation of spike times. The difference in spike-timing accuracy between repetitions of the ABAB-Same and -Var series decreased with increasing Gaussian window width, leaving no significant difference once this width had reached 98 ms (linear mixed-effect models at each time point). This suggests that sensitivity to subtle, natural variations in acoustic features requires a short time scale (< 100 ms) that fits within the duration range of individual syllables ([63.5–203.6 ms] in the present study).

### No relationship between responses and variations in auditory stimuli

To investigate the extent to which the trial-to-trial variability in spike train accuracy depended on the presence and degree of variability in syllable features, we examined how variations in syllable length contributed to the reliability of spike trains. This was achieved by employing linear time warping that allows the aligning of all spike-trains evoked by individual A and B syllables from the ABAB-Var series on the same time axis (see Methods), which reduces the variability in the alignment of syllable on- and offsets. A paired t-test on CorrCoef values from comparisons of spike trains between blocks revealed that time warping significantly changed these values (t_20_ = − 2.60, *p* = 0.017). However, this change was small, CorrCoef values changed only slightly after time warping (before: 0.081 ± − 0.01 *vs* after: 0.083 ± 0.01, mean ± STD) but significant differences remained between the ABAB-Same and -Var series (mean ± STD = 0.16 ± − 0.026; t_20_ = − 17.6, *p* < 0.0001). Variations in syllable length can therefore explain only a small part of the lower reliability of spike trains evoked by the ABAB-Var series. To assess whether acoustic differences between two variants of a given ABAB-Var series are related to the reliability of spike trains evoked by these two variants, we computed similarity scores, entropy, and pitch differences between the first and 59 subsequent sequences in ABAB-Var series using Sound Analysis Pro^21^. Duration of stimuli was computed with Avisoft-SASLabPro (v. 5.2.15). We calculated CorrCoef for the spike trains evoked by the first and by subsequent sequences of the ABAB-Var series. The similarity score, which describes the acoustic similarity of a pair of sound stimuli based on several acoustic parameters, confirmed the existence of subtle variations in the acoustic structure of syllables (mean ± SD: 96.32% ± 3.60, range: [54–100%]). Linear regression based on either similarity scores, entropy, pitch differences, or stimuli duration and spike-train reliability did not reveal any significant correlations (*p* > 0.15; Supplementary Fig. S1a-d). Thus, we found no relationship between trial-to-trial reliability of spike trains and the degree of variability in acoustic features across renditions, a result that provides additional support for the non-linear processing of acoustic features^20,26,34–36^.

### Effect of context on the repetition of the AB pair within sequences

**Figure 5:**
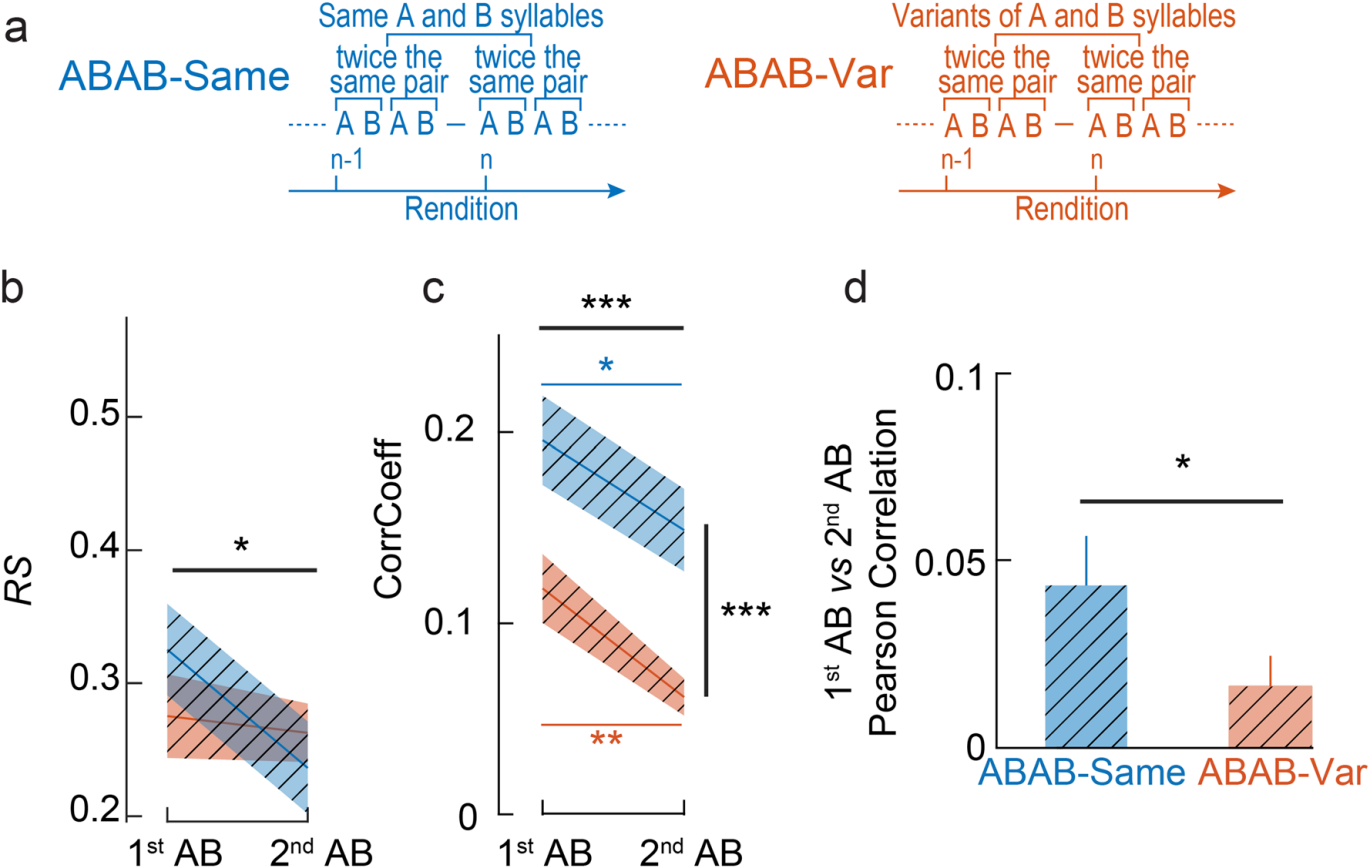
Responses to the two AB pairs that form ABAB sequences reflects sensitivity to the context in awake birds. (**a**) For ABAB-Same series, the same syllables A and B were used to build pairs of syllables AB that were repeated twice within a sequence, so two consecutive sequence renditions (n) include two repetitions of the same pairs of syllable AB. For ABAB-Var series, variants of the syllables A and B were used to build pairs of syllables AB that were repeated twice within a sequence so two consecutive sequence renditions include variants of the pairs of syllable AB repeated twice. (**b**) Strength of responses (*RS* values) changed from the first AB pair to the second one. The exposure to the first pair of syllables AB impacts the responses to the second pair of syllables AB within a stimulus rendition in awake birds (**b**–**d**). Evoked auditory responses (**b**) and CorrCoef (**c**) were overall higher for ABAB-Same than for ABAB-Var sequences and were lower for the second pair of syllables AB than for the first pair. Yet, Pearson correlation coefficient measured on each individual spike train between the first and second pair of syllables AB was lower for ABAB-Var than ABAB-Same sequences (**d**). *, ** and ***, *p* < 0.05, 0.01 and 0.001, respectively (see main text for statistics details).

NCM neurons are sensitive to syllable ordering and the auditory context in which they occur, *i.e.*, the syllables that precede the syllable of interest. Sequence stimuli used in the ABAB-Same and ABAB-Var series were all built from a given pair of AB syllables repeated twice to form an ABAB sequence. What differed between the ABAB-Same and ABAB-Var series was the auditory context in which the ABAB sequences occurred, *i.e.*, the types of sequences that preceded them. For example, the 51^st^ rendition of the ABAB sequence in the ABAB-Same series was preceded by 50 repetitions of the same ABAB sequence (so 100 times the identical pair of AB syllables) while the 51^st^ rendition of the ABAB sequence in the ABAB-Var series was preceded by 50 natural variants of the ABAB sequence. To assess whether the type of context affected responses to the second rendition of the AB pair within ABAB sequences in awake birds (Fig. 5a), we therefore analyzed the *RS* values and found a significant decrease in responsiveness with AB pair repetition within the sequences used in either series (F_1,172_ = 5.90, *p* < 0.02; Fig. 5b) but no difference between the two series (F_1,172_ = 0.32, *p* = 0.57) and no significant interaction between the two factors (F_1,172_ = 3.34, *p* = 0.07). Analyses of spike timing accuracy based on CorrCoef also showed an effect of AB pair repetition on responses (F_1,172_ = 24.42, *p* < 0.0001; Fig. 5c). Interestingly, the effect of AB pair repetition was observed for both the ABAB-Same and ABAB-Var series, indicating that even if CorrCoef for spike trains evoked by ABAB-Var were low, they could reveal changes in spike train accuracy. Hence, the temporal pattern of discharges was affected by the AB pair repetition in both contexts. However, the Pearson correlation coefficient for the trial-by-trial comparisons between the spike trains evoked by either AB pair yielded a significant difference between the ABAB-Same and -Var series (paired t-test, t_55_ = − 2.07, *p* = 0.043; Fig. 5d) with larger effects of the AB pair repetition on the temporal pattern of spike trains in ABAB-Var series. These results provide evidence that the auditory context affects NCM responses.

## Discussion

Across renditions vocal signals vary acoustically, which raises the question of whether these variations are detected and play any functional roles. Subtle, natural variations in the fine acoustic structure of song syllables can be behaviorally discriminated by adult zebra finches^11^. Our study provides evidence that these variations are encoded by neurons in a high-level auditory area where spike train reliability differs depending on whether acoustic details vary across iterations.

With regard to the functional role, we investigated the potential impact of natural variations on the adaptation of neural responses to a repeated stimulus. Such repetitions are assumed to play an important role in auditory memory formation through the binding of auditory objects, a crucial part of the auditory scene analysis^37^. To the best of our knowledge, this is the first study to test the stimulus-specific adaptation paradigm with repeated stimuli that contained natural variations. Zebra finches intensively repeat their vocalizations with slight variations across renditions. Our original hypothesis was that natural variations should prevent or at least slow down changes in auditory responses with stimulus repetition because variants are encoded as distinct stimuli. In such a case, regarding the functional role of the adaptation, any changes in the adaptation rate could be interpreted as maintaining stimulus detection despite its repetition. Furthermore, it would bestow a greater importance on each individual’s vocalizations. If the hypothesis was true, we would also expect that variations in the time course of responses to remain unaffected because NCM neurons possess a certain level of tolerance that would allow them to encode stimuli containing slight acoustic variations as the same object. Our results provide support to both predictions and thus the original hypothesis. Depending on the time scale, the impact of variations on both responses and the time course of the adaptation differed. This is consistent with studies reporting that cortical auditory neurons exhibiting stimulus-specific adaptation showed a sensitivity to auditory stimuli that operates at multiple time scales concurrently, spanning many orders of magnitude^38^.

When responses were calculated from firing rates averaged over the entire sequence duration, they showed no discernible impact of slight variations in the acoustic features of syllables. Responses decreased with stimulus repetition, as previously described in high-level auditory areas in mammals^39,40^ or songbirds^17,19,20,22,24,41,42^. Importantly, this decrease did not depend on whether the broadcast consisted of variants or identical sequences. In this study, we have reported a similar adaptation rate when greater changes in response magnitude occurred, *i.e.,* during the first presentation of an auditory stimulus. This suggests that, at the sequence duration time scale, responsive neurons encode entire sequences as a single object, irrespective of whether its syllables contain natural acoustic variations. This is consistent with previous studies that had reported no changes in the NCM auditory responses^13^, even when song stimuli were played back with an underlying environmental background noise^26^. This tolerance towards natural acoustic variations does not imply that the length of the time window over which a bird integrates acoustic information and distills it into a single object necessarily needs to match the length of the entire sequence duration. A time-series analysis of spike trains using a varying Gaussian window width yielded no statistically significant differences between the responses to playbacks of variants and identical sequences for time scales exceeding ~ 100 ms. This is consistent with previous studies who observed a peak invariance around 150 ms after the onset of different call-types in the avian auditory cortex including the NCM^13^.

Importantly, the present study also provided evidence that, at a short timescale, neuronal responses change if the acoustic features of syllables vary across renditions. Temporal reliability of spike trains was lower when the fine acoustic structure of syllables varied. Also, the time course of the spike train reliability across stimuli differed depending on whether the variant or identical sequences were played back. CorrCoef only decreased with the identical sequence. These results cannot be explained by a lack of temporal organization within spike trains evoked by playbacks of variants as this would not produce any decrease in spike-timing reliability. Although spike-train reliability was low, it remained higher than for randomly organized spike trains. Furthermore, CorrCoef decreased between the first and second AB pair (from within an ABAB sequence) even when sequence variants were used. This cannot be due to differences in firing rates as CorrCoef is independent of the firing rate^29^. Moreover, the firing rate decreased in a similar fashion as a result of stimulus repetition even when sequences varied acoustically across renditions. We therefore propose that the temporal resolution of spike trains depends on whether variants or identical sequences are used as stimuli. We compute the reliability coefficient for each spike-train by varying the Gaussian window width from 1 to 200 ms. Interestingly, CorrCoef plateaued at about 10 ms when the identical sequence was used as a repeated stimulus, *i.e.*, the temporal precision of spike trains evoked by identical sequences was in a time scale of about 10 ms. In contrast, no clear plateau was reached when using varying sequences for any of the tested window widths.

The non-linear integration of acoustic information by NCM neurons makes it difficult to interpret their auditory responses, as exemplified by their adaptative responses to stimulus repetition^20,26,43^. Unsurprisingly, we therefore did not find any significant correlation between the temporal patterns of the spike train acoustic measures (pitch, entropy, similarity score, and duration). Neither one nor a combination of acoustic features directly contributed to the neuronal auditory responses in a high-order brain area which suggests that these responses may be context-sensitive^20,26,43^. For example, the order of syllables within songs affected the neuronal response to individual song syllables, *i.e.*, the neuronal activity depended on which syllable immediately preceded the current syllable^20^. Here, we employed two series types, namely ABAB-Same and ABAB-Var, which allowed us to examine the impact of global context. The differences observed in the temporal patterns of spike trains between the first and the second pair as a result of differing contexts, lends support to the idea that neuronal responses in NCM reflect a long-term integration of auditory information that exceeds several hundreds of milliseconds, *i.e.,* the time period between two consecutive ABAB sequences. Therefore, NCM neurons are not only sensitive to the fine acoustic structure of syllables but also to the global context in which they occur. This is consistent with previous observations of information being integrated across a range of time scales in the auditory cortex and other cortical areas of both humans^44^ and non-human mammalian species^38,45,46,for a review47^. Here, we use the term of temporal integration scale to mean the time window during which neurons are sensitive to auditory stimuli, which differs from the time window size that yields the best discrimination between auditory stimuli.

Finally, by employing different types of neural computations based on distinct temporal integration periods, NCM may provide neural mechanisms to extract critical perceptual information. We propose the existence of three such integration periods, each with a different purpose: (i) a relatively short period to provide precise temporal information, (ii) an intermediate period to allow categorization of sound stimuli, and (iii) a rather long period to provide comprehension of the global context in which sounds occur. The duration of these integration periods can be related to the richness of behaviorally relevant information encoded in vocal signals, calls and songs^11,48–50^, and to the richness of their temporal structure over multiple time scales^51,52^ such as in music and speech^53^. A hypothesis based on multiple time integration periods has already been proposed for speech and, beyond that, as a general mechanism for audition^44,54,55^.

In summary, our study has shown that neurons in a non-primary cortex-like auditory region were sensitive to fine, natural, acoustic variations in song elements as well as the context in which the song elements occurred, suggesting that auditory information is integrated over a range of time scales.

## Methods

### Subjects and housing conditions

Data were collected on eleven adult male zebra finches (*Taeniopygia guttata*) reared socially in the breeding colony of the Paris-Saclay University. Birds were kept under a 12:12 light–dark cycle, with food and water ad libitum, and an ambient temperature of 22–25 °C.

All experiments were performed in accordance with national (JO 887–848) and European (86/609/EEC) legislation on animal experimentation and under national license 05186.01 (project 2015–3 approved by the Ethics Committee no. 59 [CEEA (Comité d’Ethique en Expérimentation Animale) Paris Centre et Sud]). Methods used in this study followed the ARRIVE guidelines on animal experiments^56^.

### Auditory stimuli

Zebra finch song syllables can be categorized into distinct syllable types. To build auditory stimuli, we first selected song syllable types from our collection of song bouts previously recorded (sampling rate: 32 kHz) from adult male zebra finches that had lived in the laboratory’s aviary for years before the experiment. Birds used in the present study had never been exposed to these songs prior to the electrophysiological investigation. A total of 81 syllable types and 60 renditions of each of them were extracted from the bird’s repertoire of twelve male zebra finches. Note that we selected any type of song syllables, so including harmonics and more noisy song syllables. From this dataset, we chose two distinct syllable types, called ‘A’ and ‘B’, that could have been sung by a single or two individuals, to form ABAB sequence stimuli of 0.70 ± 0.30 s duration with 30–50 ms as inter-syllable silence intervals, as typically found in zebra finch songs. Syllable duration ranged from 57 to 235 ms (mean ± SD: 134.2 ± 39.6). Then, we built ABAB-Same series that each consisted of 60 repetitions of a given ABAB sequence (see an example of an ABAB sequence stimulus, called A_1_B_1_A_1_B_1_, composed of harmonic syllables, in Fig. 1) and ABAB-Var series that each consisted of 60 variants of a given ABAB sequence (example composed of noisy syllables; note that other ABAB-Var sequences included harmonic syllables). Variants were labelled as from A_1_B_1_A_1_B_1_ to A_60_B_60_A_60_B_60_ (Fig. 1). Seven ABAB-Same series and eight ABAB-Var series were built. We used Sound Analysis Pro 2011^21^ to compute the accuracy score (Fig. 1c), which provides a fine-grained quantification of the acoustic similarity, between each renditions of the A and B syllables for each sequences of the ABAB-Same and ABAB-Var series, *i.e.* syllables A *vs* A, B *vs* B, A *vs* B. For the ABAB-Same series for which syllables A and B within a sequence were always the same, an ANOVA revealed a significant difference of the average accuracy scores of the syllables (F_2,28_ = 222.9, *p* < 0.001) and a post-hoc Tukey HSD multiple comparison analysis revealed that it was significantly lower for syllables A *vs* B (average accuracy score = 73.5%) than for syllables A *vs* A (100%) and B *vs* B (100%). For the ABAB- Var series, for which there were 60 variants of the A and B syllables, an ANOVA revealed a significant difference of the average accuracy scores of the syllables (F_2,25_ = 13.93, *p* < 0.001) and a post-hoc Tukey HSD multiple comparison analysis revealed that it was significantly lower for syllables A *vs* B (average accuracy score = 69.2%) than for syllables A *vs* A (82.8%) and B *vs* B (82.4%). None of the ABAB sequences used to build ABAB-Same series were used in ABAB-Var series. All sequences in both series types started with the same introductory note. When a series was played back, sequence stimuli were delivered at a rate of one per second.

### Electrophysiological recordings

Neuronal activity in NCM was recorded in awake (n = 4) and in anesthetized (n = 7) adult male zebra finches while presenting at least one ABAB-Same and one ABAB-Var series.

### Acute recordings

Birds were anesthetized with isoflurane gas (in oxygen; induction: 3%, maintenance: 1.5%) that flowed through a small mask over the bird’s beak. The bird was immobilized in a custom-made stereotaxic holder that allowed the head to be tilted at 45° and placed in a sound attenuation chamber. Lidocaine cream was applied to the skin. A window was opened in the inner skull layer and small incisions were made in the dura. A multi-electrode array of eight or 16 tungsten electrodes (1–2 MΩ impedance at 1 kHz; Alpha Omega Engineering, Nazareth, Israel) that consisted of two rows of four or eight electrodes separated by 100 μm apart, with 100 μm between electrodes of the same row was lowered to record extracellular activity. The array was positioned 0.3**–**0.5 mm lateral and 0.7**–**0.9 mm rostral to the bifurcation of the sagittal sinus in either the left or the right hemisphere, with a micromanipulator, as in previous studies^15,16,20,22^. The probe was lowered very slowly until electrode tips reached 1200 μm below the brain surface. From 1200 to 2000 μm below the brain surface, auditory stimuli were delivered when the amplitude of action potential waveforms recorded with at least one of the eight electrodes was clearly distinct from background noise. Recording sites were at least 100 μm apart to minimize the possibility that the neural activity recorded from two successive sites originated from the same single units. Electrode signals were amplified and filtered (gain 10,000; bandpass: 0.3–10 kHz; AlphaLab SnR, AlphaOmega LTD) to extract multi-unit activity. During recordings, voltage traces and action potentials were monitored in real time using the AlphaLab SnR software. Auditory stimuli were concomitantly recorded and digitized to precisely determine the onset of NCM responses with respect to the sound stimulus. While spiking activity was recorded, auditory stimuli were broadcasted through a loudspeaker situated 30 cm from the bird’s head. We played back one ABAB-Same and one ABAB-Var series. From one recording site to the following one, because of the habituation phenomenon in NCM, we changed the set of series used as auditory stimuli and the order of series. All stimuli had been normalized to achieve maximal amplitude of 70 dB (Audacity software) at the level of the bird’s head. Spike sorting of neuronal activity was done offline (see below).

### Chronic recordings

Procedure for chronic recordings were similar as described previously^20^. In short, we used a custom build screw microdrive that allows a microelectrode array to be dorsally repositioned. We used arrays of eight electrodes (two rows of four electrodes separated by 100 μm apart; with a ground silver wire and a reference wire; 1–2 MΩ impedance at 1 kHz; Alpha Omega Engineering, Nazareth, Israel). Once the array was lowered into the brain to a depth of 1200 μm and the reference wire inserted between the outer and the inner skull layers, the microdrive was secured to the skull using dental cement. Recordings started after a few days of recovery. Before a recording session, we rotated the screw by ½ turn to advance the microelectrode array in step as ~ 100 microns. At least 24 h separated two recording sessions. From one recording session to the following one, we changed the set of series used as auditory stimuli. Birds were restrained with a jacket around their body during the recordings sessions that were performed in a sound-attenuation chamber (inner dimensions: L80xH70xP70 cm) with a loudspeaker horizontally located at 30 cm from their head.

### Data processing and analysis

In anesthetized birds, spike sorting was performed using the template-matching algorithm of the Spike2 software (version 8.0, Cambridge Electronic Design, CED, Cambridge, UK). NCM contains at least two populations of neurons that can be distinguished on the width of the spike waveform and the firing rate^20,26,27^, so restricted our analyses to well-isolated units. In awake birds, neural traces of multiunit activity were subjected to amplitude thresholding spike detection. Responses to stimuli were quantified by calculating averaged firing rates during sequence presentation and by computing the *RS* index^15,22,57^. The *RS* index was calculated by subtracting the spontaneous firing rate (*B*_*FR*_) from the evoked firing rate (*E*_*FR*_) and then by dividing this value by their sum:

$$RS= \frac{{E}_{FR}- {B}_{FR}}{{E}_{FR}+{B}_{FR}}$$

*RS* values fall between + 1 and − 1, where values > 0 indicate an excitatory response and values < 0 indicate an inhibitory response. The *B*_*FR*_ was measured over the 200 ms period preceding the stimulus onset. We calculated *RS* values for the 60 renditions of sequence stimuli and per block of 10 presentations, giving us 6 values per series (one per block of ten iterations of the stimulus). Note that for the ABAB-Var series, each block includes 10 variants of the auditory stimuli. Auditory responses to a stimulus in NCM decrease rapidly with stimulus repetition. To examine whether the stimulus-specific adaptation differed between ABAB-Same and -Diff series, we computed a stimulus-specific adaptation rate from responses (*E*_*FR*_*)* to the 10 first stimulus renditions by extracting the slope of the linear regression for each unit^17,23–25^.

The temporal pattern of responses evoked by both types of songs was quantified by calculating the spike-timing reliability coefficient (CorrCoef), which was used to quantify the iteration-to-iteration reliability of responses. It was computed a) per block of ten stimulus iterations and b) per iteration: it corresponds to the normalized covariance between each pair of action potential trains and was calculated as follows:

$$CorrCoef=\frac{1}{N(N-1)}\sum_{i=1}^{N-1}\sum_{j=i+1}^{N}\frac{\sigma {x}_{i}{x}_{j}}{\sigma {x}_{i}{x}_{j}\mathrm{^{\prime}}}$$

where *N* is the number of iterations, and *xixj* is the normalized covariance at zero lag between spike trains *xi* and *xj*, where *i* and *j* are the iteration numbers. Spike trains *xi* and *xj* were previously convolved with a width of the Gaussian window ranging from 1 to 200 ms. In the present study, most analyses were based on CorrCoef values calculated from a convolution with a 10 ms Gaussian window width^20^. The CorrCoef was used because this index is not influenced by fluctuations of firing rate (Gaucher et al.^29^). Note that we also computed CorrCoef values from spikes trains after performing a random permutation of the time at which occurred individual spikes during each stimulus rendition. This random permutation thus gave us an estimation of the CorrCoef when spikes timing is randomly distributed.

Spike-timing reliability might be impacted by the variation of syllables’ duration across each rendition of the ABAB-Var sequences. Given that, we performed a linear time warping of each syllable so that all renditions of an ABAB-Var sequence were aligned on the same time axis^58^. Syllable boundaries were automatically detected according to the threshold crossing of the root-mean square of the amplitude of each rendition. We extracted the maximum duration of A and B syllables within the sequence and used it as a reference timing. We then linearly stretched or compressed each syllable to match its duration to the maximum duration of its reference. Each individual spike train was then projected to the time warped axis of the corresponding syllable. This algorithm thus reduces the temporal variation of the spike trains from one trial to another.

To examine whether CorrCoef values depended on acoustic variability from one variant to another, we quantified differences in acoustic features and degree of similarity between all variants used to build a given ABAB-Var series with SAP 2011^21^. From CorrCoef values computed from spike trains evoked by the two variants used in comparisons, we performed linear regressions.

Statistical computations were carried out in R (4.0.2) and MATLAB (2020a). Firing rates, *RS* and CorrCoef values were analyzed using either repeated measures (RM) ANOVA in Linear Mixed Models (R package ‘nlme’ version 3.1-152) or paired T-tests (R package ‘stats’ version 4.1.0). Depending on the analysis, the block repetition (n = 6), the series type (ABAB-Same *vs*. ABAB-Var) and/or AB pair identity (the first *vs*. the second one) were included as cofactors in the model. We used planned contrast and least-square means adjusted with the Tukey HSD tests for assessing pair-wise differences (emmeans function from R package ‘emmeans’ version 1.6.1).

### Histology

At the end of each experiment, the animal was euthanized with a lethal dose of pentobarbital and the brain quickly removed from the skull and placed in a fixative solution (4% para-formaldehyde). Sections (100 μm) were cut on a vibratome to examine the location of multielectrode array penetration tracks.

## Supplementary Information

Supplementary Figure 1.

Supplementary Legends.

## Data Availability

Data will be made available upon reasonable request.
