# Supplementary figures and images for: Distinct timescales for the neuronal encoding of vocal signals in a high-order auditory area
Source: Sci Rep. 2021 Oct 4;11:19672. doi: 10.1038/s41598-021-99135-w (PMC8490347; doi:10.1038/s41598-021-99135-w)

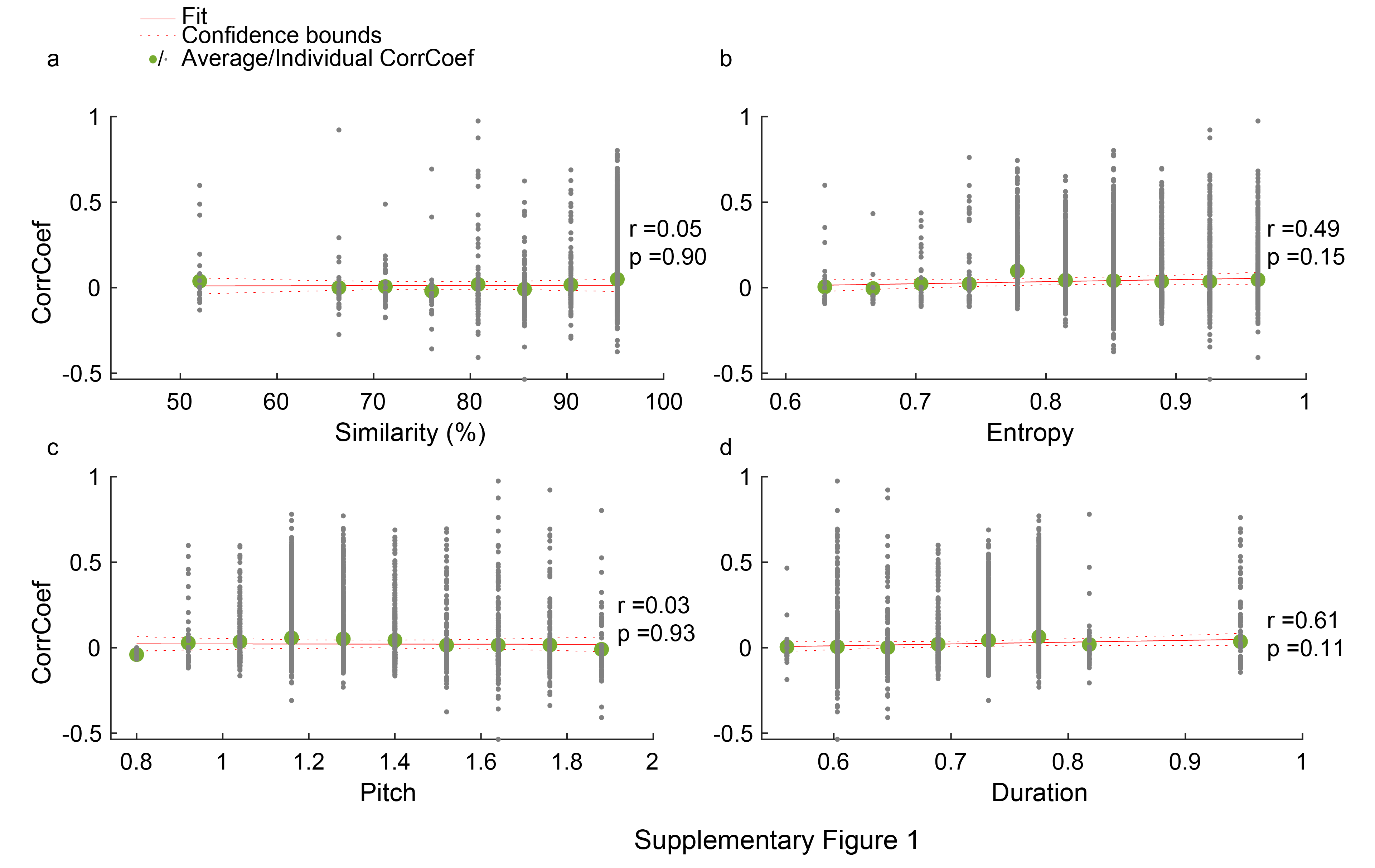

Supplement: Supplementary file 1 — Supplementary Figure 1. [file 41598_2021_99135_MOESM1_ESM.png]
