# Supplementary material for: Distinct timescales for the neuronal encoding of vocal signals in a high-order auditory area
Source: Sci Rep. 2021 Oct 4;11:19672. doi: 10.1038/s41598-021-99135-w (PMC8490347; doi:10.1038/s41598-021-99135-w)
Supplement: Supplementary file 2 — Supplementary Legends. [file 41598_2021_99135_MOESM2_ESM.docx]

Supplementary Figure 1: Acoustic feature variability does not correlate with spike timing reliability. Linear regression between differences in similarity scores (a), entropy (b), pitch (c) and stimuli duration (d) from the first sequence exemplar of the ABAB-Var series and one of the 59 following ones *vs* CorrCoef values, computed from the spike train evoked by the first sequence rendition and one of the 59 following ones, the same as used to quantify acoustic differences. The thick line represents the slope of the regression; Pearson’s r and *p* values on each plot; green dot: averaged CorrCoef values.
